# Lysophosphatidylinositol Induced Morphological Changes and Stress Fiber Formation through the GPR55-RhoA-ROCK Pathway

**DOI:** 10.3390/ijms231810932

**Published:** 2022-09-18

**Authors:** Keisuke Nakajima, Saori Oka, Takashi Tanikawa, Yoko Nemoto-Sasaki, Naoki Matsumoto, Hiroki Ishiguro, Yoichiro Arata, Takayuki Sugiura, Atsushi Yamashita

**Affiliations:** 1 Faculty of Pharma-Science, Teikyo University, 2-11-1 Kaga, Itabashi-ku, Tokyo 173-8605, Japan; 2 Pharmaceutical Department, Teikyo University Hospital, 2-11-1 Kaga, Itabashi-ku, Tokyo 173-8606, Japan; 3 Faculty of Pharmacy and Pharmaceutical Sciences, Josai University, 1-1 Keyakidai, Sakado 350-0295, Japan; 4 Department of Clinical Genetics, Graduate School of Medicine, University of Yamanashi, 1110, Shimokato, Chuo-shi 409-3821, Japan

**Keywords:** lysophosphatidylinositol, GPR55, endocannabinoid, lysophospholipid mediator, morphological change, G_12/13_-RhoA-ROCK pathway

## Abstract

We previously reported that lysophosphatidylinositol (LPI) functions as an endogenous agonist of GPR55, a novel cannabinoid receptor. However, the physiological roles of LPI-GPR55 have not yet been elucidated in detail. In the present study, we found that LPI induced morphological changes in GPR55-expressing HEK293 cells. LPI induced the cell rounding of GPR55-expressing HEK293 cells but not of empty-vector-transfected cells. LPI also induced the activation of small GTP-binding protein RhoA and increased stress fiber formation in GPR55-expressing HEK293 cells. The inhibition of RhoA and Rho kinase ROCK by the C3 exoenzyme and the ROCK inhibitor reduced LPI-induced cell rounding and stress fiber formation. These results clearly indicated that the LPI-induced morphological changes and the assembly of the cytoskeletons were mediated through the GPR55-RhoA-ROCK pathway.

## 1. Introduction

Δ^9^-Tetrahydrocannabinol (Δ^9^-THC) is one of the major active substances of marijuana [1]. Most of the pharmacological actions of Δ^9^-THC have been shown to be mediated by two types of G-protein-coupled receptors (GPCRs), CB1 and CB2 receptors [2,3]. These receptors share 44% overall identity (68% identity for the transmembrane domains). The CB1 receptor is abundantly expressed in the nervous system, including the brain. The psychoactive effects of Δ^9^-THC, including altered perception, euphoria, hallucinations, and enhanced appetite, are mediated by the CB1 receptor. In contrast, the CB2 receptor is abundantly expressed in several types of leukocytes, such as macrophages/monocytes, B lymphocytes, and natural killer cells, and is assumed to participate in the regulation of immune responses and/or inflammatory reactions. We found that 2-arachidonoylglycerol (2-AG) was an endogenous cannabinoid receptor ligand for CB1/CB2 receptors [4,5,6,7].

LPI is an “old” lysophospholipid mediator that is proposed to exhibit several biological activities, including insulin secretion via the mobilization of Ca^2+^ and cancer proliferation [8,9,10,11,12]. However, since the specific receptor of LPI had not yet been identified, its mechanisms of action had not been fully established for long time [13,14]. 

Human GPR55 was initially identified as orphan GPCR, which is abundantly expressed in the brain [15]. The human GPR55 gene is mapped to chromosome 2 q37 and encodes a protein of 319 amino acids. GPR55 is expressed in several mammalian tissues, such as breast adipose tissue, the testis, spleen, and several regions of the brain [13,14,16]. GPR55 was proposed to function as a cannabinoid receptor [13,14,17,18,19,20,21]. Of note, GPR55 has low sequence identity with the CB1 receptor (13.5%) and the CB2 receptor (14.4%), respectively [2,3,8,13].

We subsequently identified LPI, particularly, 2-arachidonoyl LPI, as an endogenous ligand for GPR55 through the activation of extracellular signal-regulated kinase (ERK) and a rapid transient increase in intracellular free Ca^2+^ ([Ca^2+^]_i_) in GPR55-expressing HEK293 cells [22,23,24]. However, even with our and other investigators’ efforts, the physiological and/or pathophysiological significance of LPI-GPR55 has not yet been elucidated in as much detail as that of other lysophospholipid mediators, such as LPA and S1P [13,14,15,16,17,18,19,20,21,22,23,24,25,26,27,28,29].

In the present study, we examined LPI-triggered GPR55-dependent morphological changes in cells. We found that LPI induced cell rounding and stress fiber formation in GPR55-expressing HEK293 cells. Such cell rounding may be involved in the rearrangement of neural networks during neurite retraction. Rounding may be also involved in the movement of white blood cells such as chemotaxis. LPI-induced morphological changes were mediated by the activation of small GTP-binding protein RhoA and Rho kinase ROCK. We also found that the different signaling pathways ran downstream of LPI-GPR55 to several destinations and compared those to cell rounding and to the activation of ERK and increases in [Ca^2+^]_i_. Furthermore, the physiological significance of LPI-induced morphological changes in cells was discussed. 

## 2. Results

### 2.1. 2-Arachidonoyl LPI Induced Cell Rounding in GPR55-Expressing Cells

**Figure 1:**
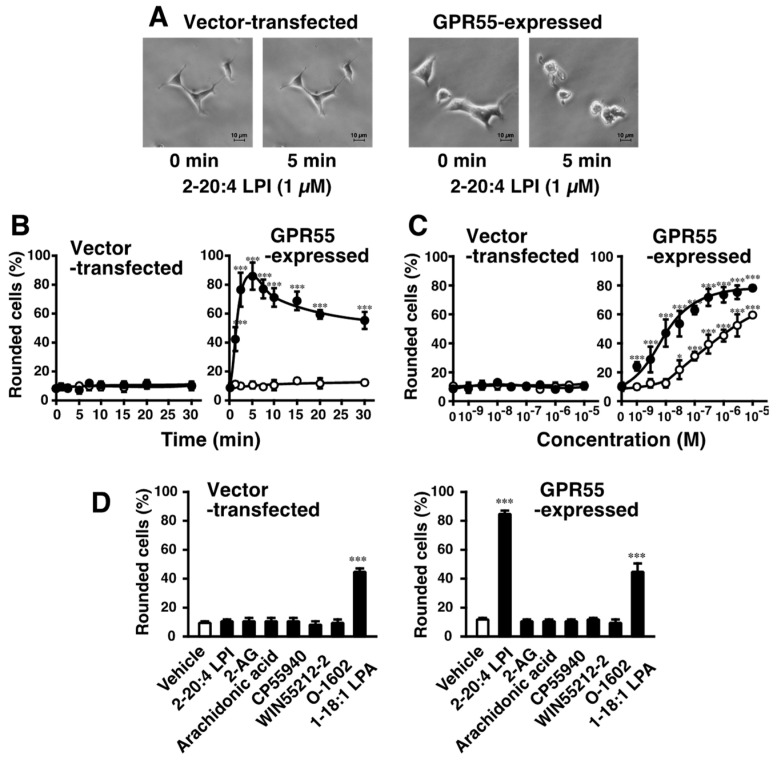
Effects of 2-arachidonoyl LPI on cell rounding of GPR55-expressing HEK293 cells. (**A**) Morphological changes induced by 2-arachidonoyl LPI. Vector-transfected cells (left) or GPR55-expressing cells (right) were challenged with 2-arachidonoyl LPI (1 µM) at 37 °C for 0 or 5 min. Scale bar, 10 μm. The results are representative of three independent experiments, which gave similar results. (**B**) Time course of cell rounding. Vector-transfected cells (left) or GPR55-expressing cells (right) were treated with the vehicle (DMSO; open circles) or 2-arachidonoyl LPI (1 µM; closed circles) at 37 °C for the indicated periods. (**C**) Dose-dependent effects of LPI on cell rounding. Vector-transfected cells (left) or GPR55-expressing cells (right) were treated with the indicated concentrations of 1-stearoyl LPI (closed circles) or 2-arachidonoyl LPI (closed circles) at 37 °C for 5 min. (**D**) Effects of various cannabinoids and related compounds. Vector-transfected cells (left) or GPR55-expressing cells (right) were treated with the indicated compounds (1 µM) at 37 °C for 5 min. After treatment, images of cells were obtained, and cell rounding was analyzed. Statistical analyses were performed with one-way analysis of variance (ANOVA), followed by Dunnett’s multiple comparison test (**B**) or Tukey–Kramer multiple comparison test (**C**,**D**). The values are means ± SDs of five determinations. * *p* < 0.05, *** *p* < 0.001 versus values at 0 min (**B**) and versus control values obtained with 0 nM LPI (**C**,**D**).

2-arachidonoyl LPI challenge induced morphological changes in GPR55-expressing HEK293 cells. 2-Arachidonoyl LPI (1 µM) induced the cell rounding of GPR55-expressing HEK293 cells (Figure 1A). The 2-arachidonoyl LPI-induced cell rounding of GPR55-expressing HEK293 cells occurred in a time-dependent manner, and maximal effects were observed after approximately ~5 min (Figure 1B). Approximately 80% of cells were rounding during the 5 min incubation with 2-arachidonoyl LPI. After the maximal effect, the rounding of cells gradually returned to the original morphology (~60 min). In contrast, cell rounding was not observed in empty-vector-transfected cells (Figure 1A,B). 

2-Arachidonoyl LPI-induced cell rounding was dependent on the concentration of LPI (Figure 1C, right). Even a high concentration of 2-arachidonoyl LPI did not induce the rounding of empty-vector-transfected cells (Figure 1C, left). 

**Figure 2:**
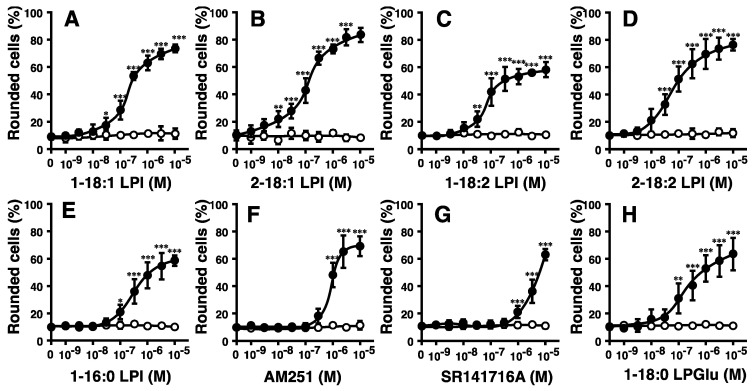
Effects of various species of LPI and related molecules on cell morphology in HEK293 cells stably expressing GPR55. Vector-transfected cells (open circle) or GPR55-expressing cells (closed circle) were challenged with various concentrations of 1-oleoyl LPI (**A**), 2-oleoyl LPI (**B**), 1-linoleoyl LPI (**C**), 2-linoleoyl LPI (**D**), 1-palmitoyl LPI (**E**), AM251 (**F**), SR141716A (**G**), or 1-stearoyl LPGlu (**H**) at 37 °C for 5 min. After treatment, images of cells were obtained, and cell rounding was analyzed. Statistical analyses were performed with one-way analysis of variance (ANOVA), followed by Dunnett’s multiple comparison test. The values are means ± SDs of five determinations. * *p* < 0.05, ** *p* < 0.01, and *** *p* < 0.001 versus values with vehicle alone (*n* = 5).

The potencies of various molecular species of LPI markedly differed (Figure 1C and Figure 2A–C). The highest level of activity was observed with 2-arachidonoyl(20:4) LPI (Figure 1C, right) at an EC_50_ of 10 nM. Its activity was approximately 30-fold stronger than that of 1-stearoyl(18:0) LPI at an EC_50_ of 300 nM (Figure 1C, right). The rank order of potency was 2-arachidonoyl(20:4) > 2-linoleoyl(18:2) = 2-oleoyl(18:1) > 1-oleoyl(18:1)> 1-linoleoyl(18:2) > 1-stearoyl(18:0) = 1-palmitoyl(16:0). The maximal effects of 1-acyl LPIs (1-palmitoyl, 1-stearoyl, 1-linoleoyl, and 1-oleoyl LPIs) were weaker than those of 2-acyl LPIs, including 2-arachidonoyl LPI, suggesting that 1-acyl LPIs acted as a weak partial agonist (Figure 1C and Figure 2A–E). 

### 2.2. Effects of Various Cannabinoid Ligands on GPR55-Dependent Cell Rounding 

Since GPR55 was previously identified as another type of cannabinoid receptor [13,14,16,17,18,19,20,21], the effects of several cannabinoid ligands were examined (Figure 1D). 2-AG, the endogenous agonist for CB1 and CB2 receptors, did not induce the cell rounding of GPR55-expressing HEK293 cells. CP55940 (a potent agonist of CB1 and CB2 receptors), WIN55212-2 (a potent agonist of CB1 and CB2 receptors), and O-1602 (an analog of cannabidiol) were also shown to be inactive. Free arachidonic acid was inactive, suggesting that the activity of 2-arachidonoyl LPI was not mediated by the degradation and conversion of prostaglandins/leukotrienes. 1-Oleoyl LPA was shown to have activity, but the other receptor(s) were mediated, including endogenous LPA1 receptor in HEK293 cells, since activity was also observed in empty-vector-transfected cells.

AM251 and SR141716A are inverse agonists of the CB1 receptor but also function as agonists of GPR55 [6,10]. AM251 and SR141716A induced the cell rounding of GPR55-expressing HEK293 cells (Figure 2F,G). This ability was dependent on GPR55, because cell rounding induced by AM251 and SR141716A was not observed in empty vector-transfected cells.

Lysophosphatidylglucose (LPGlu) functions as an agonist of GPR55 [30]. 1-Stearoyl LPGlu induced the cell rounding of GPR55-expressing HEK293 cells (Figure 2H). Its potency was weaker than that of 2-arachidonyl LPI.

### 2.3. Involvement of RhoA and ROCK in 2-Arachidonoyl LPI-Induced and GPR55-Dependent Cell Rounding and Stress Fiber Formation

**Figure 3:**
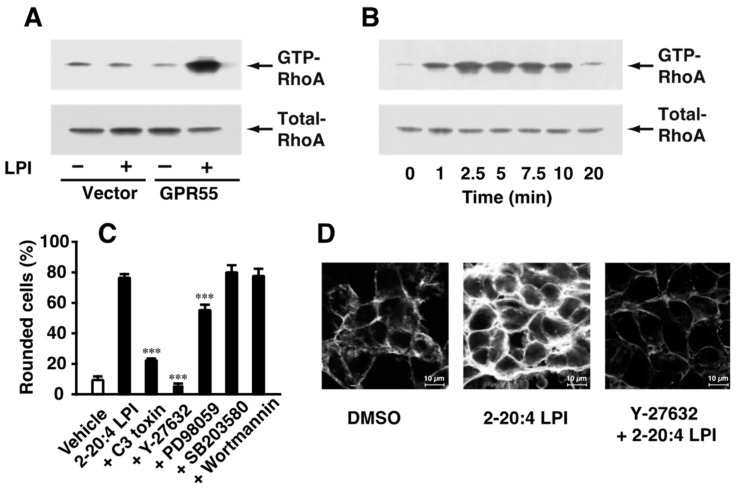
Involvement of the activation of RhoA-ROCK in 2-arachidonoyl LPI-induced and GPR55-dependent cell rounding and stress fiber formation. (**A**) Activation of RhoA. Empty-vector-transfected or GPR55-expressing HEK293 cells were challenged with vehicle (DMSO) or 2-arachidonoyl LPI (1 µM) at 37 °C for 5 min. (**B**) Time course of RhoA activation. GPR55-expressing cells were challenged with 2-arachidonoyl LPI (1 µM) at 37 °C for the indicated periods. (**C**) Effects of various inhibitors on 2-arachidonoyl LPI-induced RhoA activation. GPR55-expressing cells were pretreated with vehicle, C3 toxin (20 µg/mL, 24 h), Y-27632 (20 µM, 1 h), wortmannin (500 nM, 1 h), PD98059 (20 µM, 1 h), or SB203580 (20 µM, 1 h) and then with 1 µM 2-arachidonoyl LPI for 5 min. Statistical analyses were performed with one-way analysis of variance (ANOVA), followed by Tukey–Kramer multiple comparison test. The values are means ± SDs of five determinations. *** *p* < 0.001 versus 2-arachidonoyl LPI alone. (**D**) 2-Arachidonoyl LPI-induced stress fiber formation. Vehicle or Y-27632 (20 µM, 1 h)-treated GPR55-expressing cells were challenged with 1 µM 2-arachidonoyl LPI for 5 min. Scale bar, 10 μm. The results are representative of three independent experiments, which gave similar results (**A**,**B**,**D**).

The mechanisms underlying 2-arachidonoyl LPI-induced GPR55-dependent cell rounding were investigated. The activation of RhoA during cell rounding was examined. The activation of RhoA was detected by the precipitation of the activated GTP-binding form of RhoA with Rhotekin beads. As demonstrated in Figure 3A, the challenge with 2-arachidonoyl LPI (1 µM) induced the activation of RhoA in GPR55-expressing HEK293 cells; however, the 2-arachidonoyl LPI (1 µM)-induced activation of RhoA was not observed in empty-vector-transfected cells. The activation of RhoA was detectable 1 min after the stimulation, reached a peak after approximately 2.5–5 min up to approximately 7.5 min, and then reverted to basal levels (Figure 3B).

The effects of various inhibitors on 2-arachidonoyl LPI-induced and GPR55-dependent cell rounding were examined (Figure 3C). Cell rounding induced by 2-arachidonoyl LPI was markedly reduced by treatment with C3 toxin (the RhoA inhibitor) or Y-27632 (the ROCK inhibitor). A slight inhibitory effect was observed following pretreatment with PD98059 (the MEK inhibitor). However, pretreatment with wortmannin (PI3K inhibitor) or SB203580 (p38 MAPK inhibitor) did not affect 2-arachidonoyl LPI-induced cell rounding. Stress fibers were formed due to challenge with 2-arachidonoyl LPI, and this was potently inhibited by pretreatment with Y-27632, the ROCK inhibitor. These results clearly indicated that the activation of the RhoA-ROCK pathway was involved in 2-arachidonoyl LPI-induced cell rounding and stress fiber formation.

### 2.4. 2-Arachidonoyl LPI Induced the Activation of ERK and Increases in [Ca^2+^]_i_ in GPR55-Expressing Cells

We previously reported that LPI induced the activation of ERK and increases in [Ca^2+^]_i_ in a GPR55-dependent manner [22,23]. The signaling pathways from LPI-GPR55 to several destinations were compared. The 2-arachidonoyl LPI (1 µM)-induced phosphorylation of ERK was almost completely inhibited by pretreatment with U-0126 (MEK inhibitor). This result was reasonable, because MEK isoforms are known to function immediately upstream of ERK MAP kinases. Pretreatment with Y-27632 (ROCK inhibitor) and calphostin C (PKC inhibitor) partially inhibited the LPI-induced phosphorylation of ERK. However, the inhibitory effects of pretreatment with wortmannin (PI3K inhibitor) or herbimycin A (tyrosine kinase inhibitor) were weak. U-73122 (PLC inhibitor) did not inhibit the effects of 2-arachidonoyl LPI.

In contrast, the 2-arachidonoyl LPI-induced increase in [Ca^2+^]_i_ in GPR55-expressing cells was partially inhibited by pretreatment with herbimycin A (tyrosine kinase inhibitor), U-0126 (MEK inhibitor), or U-73122 (PLC inhibitor). Y-27632 (ROCK inhibitor), wortmannin (PI3K inhibitor), and calphostin C (PKC inhibitor) did not inhibit the Ca^2+^ response induced by 2-arachidonoyl LPI.

These results indicated that different signaling pathways were involved in each destination.

## 3. Discussion

G-protein coupled receptor GPR55 was previously identified as a novel cannabinoid receptor [17,18,19,20]. Although GPR55 is abundantly expressed in the brain, previous studies reported a relationship between brain functions and the psychoactive effects of cannabinoids [15,16,17,18,19,20,21]. GPR55 responded to Δ^9^-tetrahydocannabinol [13,17,18,19,20,21,22,23]. We and other investigators investigated endogenous ligands for GPR55 [13,17,18,19,20,21,22,23]. We found for the first time that LPI, particularly 2-arachidonyl LPI, functioned as an endogenous agonist of GPR55 [22,23,24]. Other investigators also reported similar findings [31,32,33,34,35,36]. The structure–activity relationship revealed that 2-arachidonoylglycerol (2-AG), an endocannabinoid for CB1 and CB2 receptors, did not function as an agonist of GPR55 [22,23,24]. We also found that *N*-acylethanolamine, including anandamide (*N*-arachidonoylethanolamine), was not a ligand for GPR55.

However, the physiological roles of LPI-GPR55 have not yet been elucidated in detail. We found that LPI induced morphological changes, namely, cell rounding and stress fiber formation, in a GPR55-dependent manner. The results of experiments employing inhibitors suggest that the G_12/13_-RhoA-ROCK pathway is involved in LPI-induced cell rounding. We also found that LPI induced stress fiber formation. Our results are consistent with previous findings showing that the G_12/13_-RhoA-ROCK pathway is involved in the regulation of the cytoskeleton [37]. LPA-induced neurite retraction and the assembly of stress fibers have also been reported to be mediated by G_α13_ [38,39].

We examined the structure–activity relationship of LPI in detail (Figure 1 and Figure 2). The potency of LPI for inducing GPR55-dependent cell rounding was dependent on the position and type of fatty acids in LPI. The 2-acyl types of LPI were more potent than the 1-acyl types. The rank order of potency in cell rounding corresponded to those of the activation of ERK and increases in [Ca^2+^]_i_ in GPR55-expressing cells [22,23]. These findings indicated that the rank order reflected the difference in ligand recognition of GPR55.

We also examined the signaling pathway of LPI-evoked cell rounding in GPR55-expressing HEK293 cells. Cell rounding due to 2-arachidonoyl LPI was inhibited by treatment with the RhoA inhibitor (C3 toxin) or ROCK inhibitor (Y-27632) (Figure 3C). Stress fiber formation was also inhibited by pretreatment with the same ROCK inhibitor (Figure 3D). These results clearly indicated that the RhoA-ROCK pathway was activated downstream of LPI-GPR55.

**Figure 4:**
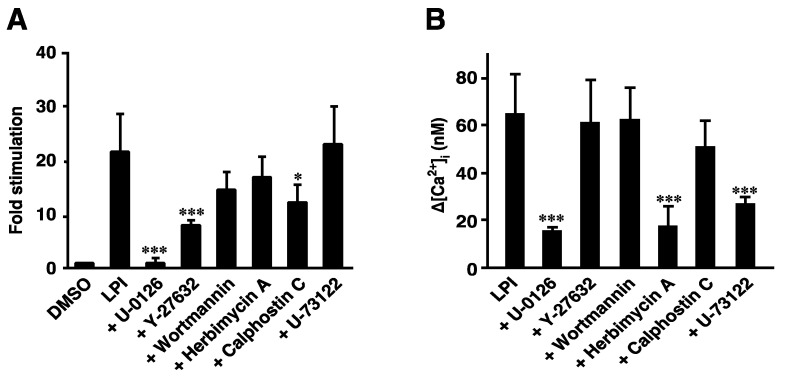
Effects of various inhibitors on the 2-arachidonoyl LPI-induced activation of ERK or increases in intracellular Ca^2+^ in GPR55-expressing cells. The 2-Arachidonoyl LPI (1 µM)-induced phosphorylation of ERK (**A**) or increases in intracellular Ca^2+^ (**B**) were measured after GPR55-expressing cells were pretreated with the indicated inhibitors. Statistical analyses were performed with one-way analysis of variance (ANOVA), followed by Tukey–Kramer multiple comparison test. The values are means ± SDs of four determinations. * *p* < 0.05 and *** *p* < 0.001 versus 2-arachidonoyl LPI alone.

We compared the signaling pathways from LPI-GPR55 to several destinations, including cell rounding, ERK activation, and Ca^2+^ responses. The activation of ERK was partly inhibited by the ROCK inhibitor (Y-27632) (Figure 4A); however, the same ROCK inhibitor completely inhibited cell rounding and stress fiber formation (Figure 3C,D). The activation of ERK was completely inhibited by the MEK inhibitor (U-0126) (Figure 4A), whereas LPI-induced cell rounding was slightly inhibited by another MEK inhibitor (PD98059) (Figure 3D), suggesting that the contribution of the MEK-ERK pathway to cell rounding was small.

In contrast, LPI-induced Ca^2+^ mobilization was not inhibited by the ROCK inhibitor (Y-27632) (Figure 4B), indicating that RhoA-ROCK was not involved in Ca^2+^ mobilization. However, the G_q_-PLC and MEK-ERK pathways were largely involved in Ca^2+^ mobilization because the PLC inhibitor (U-73122) and MEK inhibitor (U-0126) inhibited the response (Figure 4B). Although tyrosine kinase may be involved in Ca^2+^ mobilization, based on the results of experiments employing the tyrosine kinase inhibitor (herbimycin A), the contribution of tyrosine kinase to the activation of ERK is not large. The signaling pathway to the activation of ERK and Ca^2+^ mobilization partly overlapped with but was not strongly cross-linked to those of cell rounding. 

The physiological roles of the LPI-GPR55 axis in morphological changes remain unclear. Previous studies indicated that lysophospholipid mediator LPA regulated the cytoskeleton [37,38,39,40]. LPA induces the infiltration of cancer cells into mesothelial cell layers [41,42]. LPA also causes neurite retraction in neural cells [43,44]. The change in cell morphology and the rearrangement of the cytoskeleton may be involved in infiltration and neurite retraction. Cell rounding may be related to neurite retraction. We also demonstrated that LPA induced cell rounding in HEK293 cells in a GPR55-independent mechanism (Figure 1D). LPI was also previously reported to cause neurite retraction in PC12 cells [45]. GPR55 was shown to play a role in the modality-specific repulsive guidance of spinal cord sensory axons; however, the involved agonist for GPR55 was suggested to be LPGlu (Figure 2F) [30]. The present results of LPI-induced cell rounding may be related to neurite retraction and the repulsive guidance of spinal cord sensory axons by LPI or another agonist.

GPR55 and LPI may also contribute to the long-term potentiation (LTP) of hippocampal synaptic plasticity, because GPR55 was expressed in the pyramidal cells of CA1 and CA3 layers in the hippocampus and the application of LPI to hippocampal slices significantly enhanced CA1 LTP [46]. Since LPI-GPR55-dependent morphological changes gradually reverted to the original morphology (~60 min after the stimulation; related data at ~30 min are shown in Figure 1B), LPI-GPR55 may play a role in the rearrangement of the neural network. 

Jenkin et al. demonstrated that the cannabinoid receptors affected the hypertrophy of human proximal tubular cells [47,48]. AM251, an agonist on GPR55 and an inverse agonist/antagonist of CB1, reduced the hypertrophy of proximal tubular cells. In the present study, the ectopic expression of GPR55 led to induce the LPI-evoked cell rounding in HEK293 cells. Cell rounding via GPR55 activation is consistent with the reduced size of proximal tubular cells through GPR55. Since the relevance of renal hypertrophy and apoptosis was proposed, cell rounding may explain the physiological meanings of LPI/GPR55 on the functions of the kidney. Although HEK293 cells were originally derived from embryonic kidneys, our results in Figure 1 and Figure 2 indicated that the cells did not express endogenous GPR55. 

Because GPR55 is expressed in immune tissues, such as the spleen, thymus, and small intestine [16,24], morphological changes, such as cell rounding due to LPI, may be involved in immune responses. Cell rounding may be involved in the infiltration of immune cells. Another lysophospholipid messenger LPA was shown to induce T-cell motility through a RhoA-ROCK-dependent mechanism [49]. LPI-GPR55 may also be involved in the motility and infiltration of immune cells, including lymphocytes.

One unexpected result was observed. Although some investigators indicated that O-1602 and abnormal cannabidiol act as potent GPR55 agonists [50,51], we did not reproduce the result in GPR55-expressed HEK293 cells (Figure 1D). O-1602 and abnormal cannabidiol did not induce the phosphorylation of ERK and p38 MAPK or the increase in intracellular calcium in GPR55-expressing HEK293 cells [22,23,24]. We think that the elucidation and explanation of the discrepancy should be the subject of further study.

We also demonstrated that GPR55-expressing HEK293 cells were larger than empty-vector-transfected cells, even in the absence of stimulation by LPI (Figure 1A). The intrinsic activity of GPR55 may increase the size of cells. This activity may involve the negative regulation of the cytoskeleton. Furthermore, there are several mechanisms underlying increases in cell sizes. Cell sizes are associated with the activity of mitochondria [52]. The present study provides insights into the novel functions of GPR55, another cannabinoid and/or lysophospholipid receptor.

## 4. Materials and Methods

### 4.1. Chemicals

Arachidonic acid (20:4,n-6), essentially fatty acid-free bovine serum albumin (BSA), and lysophosphatidic acid (LPA) (1-oleoyl) sodium salt were obtained from Sigma (St. Louis, MO, USA). 1,2-Dipalmitoyl PI and 1,2-dioleoyl PI were from Avanti Polar Lipids, Inc. (Alabaster, AL, USA). 

CP55940 and Y-27632 were from Tocris (Bristol, UK). WIN55212-2 was obtained from RBI (Natick, MA, USA). O-1602 was obtained from Cayman Chemical Co. (Ann Arbor, MI, USA). PD98059, SB203580, and SP600215 were acquired from Calbiochem-Novabiochem (San Diego, CA, USA). Wortmannin was obtained from Wako Pure Chem. Ind. (Osaka, Japan). Clostridium botulinum C3 exoenzyme pcDNA4/TO and Lipofectamine^TM^ 2000 were from Invitrogen Life Technologies (Carlsbad, CA, USA). The anti-RhoA 26C4 mouse monoclonal antibody was from Santa Cruz Biotechnology, Inc. (Santa Cruz, CA, USA). The anti-mouse IgG horseradish-peroxidase-linked goat antibody was obtained from Medical & Biological Laboratories Co, Ltd. (Nagoya, Japan). Lipase (*Rhizopus delemar*) was acquired from Seikagaku Kogyo Co., Ltd. (Tokyo, Japan).

### 4.2. Preparation of Various Species of LPI

1-Acyl LPI was prepared by treating soybean PI with *Naja atra* phospholipase A2 (PLA2) in 50 mM Tris HCl (pH 7.4) and ether under vigorous stirring [23]. 1-Acyl-2-arachidonoyl PI was prepared by treating 1-acyl LPI with arachidonic anhydride dissolved in chloroform (ethanol-free) using 4-dimethylaminopyridine as a catalyst at 24 °C for 10 min. 1-Acyl-2-stearoyl PI was prepared using the same procedure using stearoyl anhydride. The resultant PI spices were purified with TLC because the impurities with acylation in the inositol ring were also synthesized.

Purified 1-acyl-2-arachidonoyl PI or 1-acyl-2-stearoyl PI was then hydrolyzed with *Rhizopus delemar* lipase (50 mg) in 2 mL of 50 mM acetate buffer (pH 5.6) containing 10 mM CaCl_2_ and 100 mM NaCl under vigorous stirring at 24 °C for 1 h to obtain 2-arachidonoyl LPI or 2-stearoyl LPI, respectively. 2-Oleoyl LPI or 2-linoleoyl LPI was also prepared via the treatment with the lipase of dioleoyl PI or of soybean PI. The enzyme reaction was terminated by adding 0.4 mL of 200 mM EDTA and 15 mL of chloroform:methanol (1:2, *v*/*v*). After the addition of 5 mL of chloroform and 5.6 mL of water, the resultant 2-acyl LPIs were recovered from the water–methanol layer of the Bligh and Dyer extraction mixture and purified using a Sep-Pak^TM^ cartridge.

1-Arachidonoyl LPI, 1-oleoyl LPI, or 1-stearoyl LPI was obtained via the fatty acid migration from the sn-2 to sn-1 positions under alkaline pH conditions. 2-Arachidonoyl LPI, 2-oleoyl LPI, or 2-stearoyl LPI was incubated in 100 mM Tris–HCl buffer (pH 8.8) at 24 °C for 30 min. The purity of other individual LPI samples was assessed to be above 95% using gas chromatography (GC) and liquid chromatography–mass spectrometry (LC-MS). Lipid phosphorus was determined as previously described [23].

We provide a simple and concise method for the preparation of 1-acyl or 2-acyl LPI using soybean and liver PI. 1-Palmitoyl LPI was prepared via the hydrolysis of soybean PI using *Naja naja atra* PLA2. 1-Stearyl LPI or 2-arachidonyl LPI was also prepared via the treatment with *Naja atra* PLA2 or *Rhizopus delemar* lipase of liver PI, although these were not used in the present study. The purity and the concomitants of other LPI species of soybean- and liver-derived LPI are described in Table 1. The purity was lower than chemically and enzymatically synthesized LPI, and the yield was much higher, because various concomitants were formed via acylation in the inositol ring in the chemical synthesis step using fatty acyl anhydride.

### 4.3. Cells

HEK293 cells were grown at 37 °C in Dulbecco’s modified Eagle’s medium (DMEM) supplemented with 10% fetal bovine serum, 100 U/mL of penicillin, and 100 µg/mL of streptomycin. A DNA fragment containing the entire open reading frame of the human GPR55 gene (GenBank^TM^ accession number NM_005683) was amplified from human spleen cDNA via PCR as previously described [22]. Cells were then transfected with GPR55-pcDNA4/TO or an empty vector using Lipofectamine^TM^ 2000 reagent. Stably transfected clones were selected in the presence of 100 µg/mL zeocin as previously described [22]. 

### 4.4. Analysis of Cell Rounding

Empty-vector-transfected or GPR55-expressing HEK293 cells were seeded on a 35-mm cell culture plate (2 × 10^4^ cells) and cultivated for 24 h to attach to the plate. Cells were challenged with LPIs with different fatty acids and positional isomers and were then cultivated for the indicated periods. In some cases, LPI concentrations varied and a negative control experiment was performed with vehicle (dimethylsulfoxide (DMSO); final concentration: 0.02%, *v*/*v*). In some cases, GPR55-expressing HEK293 cells were pretreated with C3 toxin (20 µg/mL, 24 h), Y-27632 (20 µM, 1 h), wortmannin (500 nM, 1 h), PD98059 (20 µM, 1 h), or SB203580 (20 µM, 1 h) before LPI challenge. After treatment, cell rounding was evaluated via microscopy, counting rounded cells per total cells in randomly selected areas. 

### 4.5. Analysis of Stress Fiber Formation 

The analysis of stress fiber formation was carried out as previously described [53]. GPR55-expressing HEK293 cells (2 × 10^4^ cells/mL) were seeded on 35 mm glass-bottomed dishes. After 24 h of incubation, cells were incubated in 1 mL of DMEM containing 20 mM HEPES-NaOH (pH 7.4) and 0.1% BSA in the presence of 2-arachidonoyl LPI (final concentration, 1 µM) at 37 °C for 5 min. In some cases, GPR55-expressing HEK293 cells were pretreated with Y-27632 (20 µM, 1 h) before LPI challenge. After washing with Tyrode’s solution containing 20 mM HEPES-NaOH (pH 7.4), cells were fixed with 3.7% paraformaldehyde in PBS for 10 min and permeabilized with 0.5% Triton X-100 in PBS for 5 min. After blocking with 1% BSA in PBS, cells were stained with Alexa Fluor 488 conjugated to phalloidin. Images were obtained using a confocal microscope (TCS-SP5; Leica, Wetzlar, Germany).

### 4.6. Measurement of RhoA Activation 

The analysis of the activation of RhoA was carried out as previously described [54]. Subconfluent HEK293 cells expressing GPR55 were incubated in 1 mL of DMEM containing 5 mM HEPES-NaOH (pH 7.4) and 0.1% BSA in the presence of LPI, various ligands, or vehicle (final concentration of DMSO: 0.02%, *v*/*v*) in 35 mm dishes at 37 °C for the indicated periods of time. Following incubation, the medium was aspirated, and cells were washed with ice-cold Tyrode’s solution containing 5 mM HEPES-NaOH (pH 7.4). The activated form (GTP-bound form) of RhoA was precipitated by the Rho-binding domain (RBD) of Rhotekin connected with GST and glutathione beads. The activated form of and total RhoA were detected via Western blotting against the anti-RhoA antibody. Each signal was visualized after the blot was incubated with the HRP-conjugated second antibody and detected using an ATTO imager with ECL reagent. 

### 4.7. Measurement of the Activation (Phosphorylation) of ERK 

The analysis of the activation of ERK was carried out as previously described [22,23,54]. GPR55-expressing HEK293 cells were incubated with LPI (final concentration, 1 µM in 0.02 % (*v*/*v*) DMSO as vehicle) in DMEM containing 5 mM HEPES–NaOH (pH 7.4) and 0.1% BSA in 35 mm dishes at 37 °C for 5 min. In some cases, GPR55-expressing HEK293 cells were pretreated with U-0126 (20 µg/mL, 1 h), Y-27632 (20 µM, 1 h), wortmannin (500 nM, 1 h), herbimycin A (20 µM, 1 h), calphostin C (20 µM, 1 h), or U-73122 (20 µM, 1 h) before LPI challenge. Following incubation, cells were harvested and washed with ice-cold Tyrode’s solution containing 5 mM HEPES–NaOH (pH 7.4). The activation of ERK was estimated via a Western blot analysis using a specific antibody for phospho-ERK (Cell Signaling Technology, MA, USA). The amount of ERK was also estimated via Western blotting using the ERK antibody (Cell Signaling Technology, MA, USA). Each signal was visualized after the blot had been incubated with the HRP-conjugated second antibody and detected using an ATTO imager with ECL reagent. Band intensity was quantified using ImageJ, and the ratio of phospho-ERK to total ERK was calculated. Data were expressed as fold stimulation (compared with vehicle alone or time 0). 

### 4.8. Measurement of Increases in [Ca^2+^]^i^

[Ca^2+^]_i_ was measured using a CAF-110 Ca^2+^ analyzer (JASCO, Tokyo, Japan) [22,23]. LPI or various ligands were dissolved in DMSO, and an aliquot (1 µL each) was added to the cuvette (final concentration of DMSO, 0.2%, *v*/*v*). DMSO (0.2%, *v*/*v*) per se did not markedly affect [Ca^2+^]_i_. In some experiments, cells were pretreated with various inhibitors before the measurement of [Ca^2+^]_i_ using LPI.

### 4.9. Statistical Analysis

All analyses were performed via a one-way analysis of variance (ANOVA) followed by Dunnett’s multiple comparison test (Figure 1B,C and Figure 2) or the Tukey–Kramer multiple comparison test (Figure 1D, Figure 3C and Figure 4). The results obtained are shown as the means ± SDs. The significance of the differences is indicated as follows: * *p* < 0.05, ** *p* < 0.01, and *** *p* < 0.001.

## 5. Conclusions

We found the LPI-evoked morphological changes (cell rounding) and assembly of the cytoskeletons in GPR55-expressing HEK293 cells through the GPR55-RhoA-ROCK pathway. It is very important that the morphological changes were observed not only in differentiated cells such as neuronal cells or immune cells but also in common cultured cells such as HEK293 cells.

## Figures and Tables

**Table 1:** The purity and the concomitants of soybean- and liver-derived LPIs. These LPIs were prepared by treating soybean or bovine liver PI with *Naja atra* PLA2 or *Rhizopus delemar* lipase under the indicated pH conditions. Values represent the means ± SDs from three independent experiments.

		1-16:0 LPI ^#^ (% ±SD)	1-18:2 LPI * (% ±SD)	2-18:2 LPI * (% ±SD)	1-18:0 LPI ^#^ (% ±SD)	2-20:4 LPI ^#^ (% ±SD)
	Parent PI	Soybean PI	Soybean PI	Soybean PI	Liver PI	Liver PI
Fatty Acid at *sn*-1 or -2	Enzyme, pH	PLA2, 7.4	Lipase, 8.8 ^$^	Lipase, 5.6	PLA2, 7.4	Lipase, 5.6
1-Palmitoyl (16:0)		67.4 ± 0.66	0.49 ± 0.22	0.08 ± 0.02	8.55 ± 0.12	0.00 ± 0.00
2-Palmitoyl (16:0)		4.06 ± 0.15	0.01 ± 0.01	0.21 ± 0.03	0.59 ± 0.02	0.10 ± 0.04
1-Stearoyl (18:0)		14.2 ± 0.83	0.10 ± 0.14	0.18 ± 0.26	82.42 ± 0.38	0.21 ± 0.05
2-Stearoyl (18:0)		0.71 ± 0.09	0.00 ± 0.00	0.01 ± 0.02	5.71 ± 0.14	0.00 ± 0.00
1-Oleoyl (18:1)		5.02 ± 0.23	8.14 ± 0.18	2.76 ± 0.28	2.51 ± 0.10	0.56 ± 0.07
2-Oleoyl (18:1)		0.18 ± 0.04	0.16 ± 0.23	7.99 ± 0.58	0.09 ± 0.01	5.21 ± 0.29
1-Linoleoyl (18:2)		7.34 ± 0.13	78.73 ± 0.28	22.31 ± 2.56	0.07 ± 0.01	0.90 ± 0.07
2- Linoleoyl (18:2)		0.32 ± 0.02	5.40 ± 0.12	57.37 ± 2.47	0.00 ± 0.00	10.08 ± 0.51
1-Linolenoyl (18:3)		0.70 ± 0.09	6.68 ± 0.29	2.59 ± 0.38	0.00 ± 0.00	0.00 ± 0.00
2-Linolenoyl (18:3)		0.01 ± 0.00	0.29 ± 0.08	6.49 ± 0.27	0.00 ± 0.00	0.02 ± 0.01
1-Arachidonoyl (20:4)		0.00 ± 0.00	0.00 ± 0.00	0.00 ± 0.00	0.06 ± 0.01	8.48 ± 0.47
2-Arachidonoyl (20:4)		0.00 ± 0.00	0.00 ± 0.00	0.00 ± 0.00	0.00 ± 0.00	74.43 ± 0.45

* LPI was used in this study. ^#^ LPI was not used in this study. ^$^ The treatment with lipase at pH 8.8 facilitated the fatty acid migration from the *sn*-2 to *sn*-1 positions of glycerol to form 1-acyl LPI.
